# Severe pincer morphology is associated with incident hip osteoarthritis: prospective individual participant data from 18 935 hips from the World COACH consortium

**DOI:** 10.1136/bjsports-2024-109595

**Published:** 2026-01-19

**Authors:** Noortje Riedstra, Fleur Boel, Michiel MA van Buuren, Harbeer Ahedi, Vahid Arbabi, Nigel K Arden, Sara J Baart, Sita M A Bierma-Zeinstra, Flavia Cicutini, Timothy Cootes, Kay M Crossley, David T Felson, Willem-Paul Gielis, Joshua J Heerey, Graeme Jones, Stefan Kluzek, Nancy E Lane, Claudia Lindner, John A Lynch, Joyce van Meurs, Andrea Britt Mosler, Amanda E Nelson, Michael C Nevitt, Edwin H G Oei, Jos Runhaar, Jinchi Tang, Harrie Weinans, Rintje Agricola

**Affiliations:** 1 Orthopedics and Sports Medicine, Erasmus MC University Medical Center Rotterdam, Rotterdam, Zuid-Holland, The Netherlands; 2 University of Tasmania Menzies Institute for Medical Research, Hobart, Tasmania, Australia; 3 University Medical Centre Utrecht, Utrecht, The Netherlands; 4 NDORMS, University of Oxford, Oxford, UK; 5 Department of Biostatistics, Erasmus MC University Medical Center, Rotterdam, The Netherlands; 6 Department of General Practice, Erasmus University Medical Centre, Rotterdam, The Netherlands; 7 Monash University, Melbourne, Victoria, Australia; 8 Manchester University, North Manchester, Indiana, USA; 9 La Trobe University—Bundoora Campus, Bundoora, Victoria, Australia; 10 Clinical Epidemiology Research & Training Unit, Boston University School of Medicine, Boston, Massachusetts, USA; 11 La Trobe Sport and Exercise Medicine Research Centre, School of Allied Health, Human Services and Sport, La Trobe University, Melbourne, Victoria, Australia; 12 Menzies Research Institute Tasmania, Hobart, Tasmania, Australia; 13 Department of Sports Medicine, University of Nottingham, Nottingham, UK; 14 Nuffield Department of Orthopaedics, Rheumatology and Musculoskeletal Sciences, University of Oxford, Oxford, UK; 15 Division of Rheumatology, Allergy and Clinical Immunology, Department of Medicine, University of California, Davis, California, USA; 16 University of Manchester— The Victoria University of Manchester Campus, Manchester, UK; 17 Department of Epidemiology and Biostatistics, University of California San Francisco, San Francisco, California, USA; 18 Erasmus MC University Medical Center Rotterdam, Rotterdam, Zuid-Holland, The Netherlands; 19 La Trobe Sport and Exercise Medicine Research Centre, La Trobe University—Bundoora Campus, Melbourne, Victoria, Australia; 20 Australian IOC Research Centre, Melbourne, Victoria, Australia; 21 Department of Epidemiology, University of North Carolina at Chapel Hill, Chapel Hill, North Carolina, USA; 22 University of North Carolina at Chapel Hill, Chapel Hill, North Carolina, USA; 23 University of California San Francisco, San Francisco, California, USA; 24 Erasmus Universiteit Rotterdam, Rotterdam, The Netherlands; 25 Department of Orthopaedics, Erasmus University Medical Center, Rotterdam, The Netherlands

## Abstract

**Objective:**

To assess the relationship between pincer morphology and incident radiographic hip osteoarthritis (RHOA) and study-specific subgroups.

**Methods:**

Hips completely free of RHOA at baseline and with follow-up within 4–8 years were drawn from the World COACH consortium. The lateral centre edge angle (LCEA) was calculated uniformly on all baseline radiographs. Moderate pincer morphology was defined as an LCEA ≥40°, and severe pincer morphology as an LCEA ≥45° in sensitivity analyses. The primary outcome was incident RHOA defined by a harmonised OA score. A logistic regression model with generalised mixed effects with three levels (within-cohort, within-person and within-hip side correlation) adjusted for age, biological sex and body mass index (BMI) was employed. Descriptive statistics are reported for age, biological sex and BMI.

**Results:**

18 935 hips from nine cohorts were included. 4894 hips (25.8%) had moderate pincer morphology. Within 8 years (mean 6.0±1.7 years), 352 hips (1.9%) developed RHOA. Moderate pincer morphology was not associated with RHOA (OR 1.15 (0.92–1.51)), whereas severe pincer morphology was significantly associated (OR 1.50 95% CI 1.05 to 2.15). Moderate pincer morphology in groups aged 40–50 (RR 2.67, 95% CI 1.43 to 4.95) and BMI ≥25 (RR 1.23 95% CI 0.98 to 1.71) had a higher risk compared with non-pincer hips. Women (RR 1.20 95% CI 0.93 to 1.56) with pincer morphology may be more at risk than men (RR 0.95 95% CI 0.57 to 1.58).

**Conclusion:**

The odds of developing RHOA within 8 years for hips with severe pincer morphology are 1.5 times higher than pincer-free hips, whereas moderate pincer morphology was not significantly associated with RHOA. Further research is necessary to uncover high risk subgroups of pincer morphology.

## Introduction

Osteoarthritis (OA) is a debilitating disease that significantly impacts quality of life.^1^ It is, therefore, essential to identify risk factors for OA, which can potentially be targeted in prevention and treatment strategies.^2–4^ Risk factors for hip OA include age, biological sex, genetics, physical workload, sporting activities and hip shape.^2 3 5–7^

Pincer morphology is a hip shape characterised by acetabular over coverage of the femoral head and is associated with femoroacetabular impingement (FAI) syndrome, a motion-related clinical disorder of the hip.^8–11^ Pincer morphology may cause repeated abutment between the proximal femur and the acetabulum during repetitive and terminal motion of the hip.^9^ Repeated impingement could lead to intra-articular damage (eg, cartilage and labral pathology), and ultimately hip OA.^8 9^

The association between pincer morphology and radiographic hip osteoarthritis (RHOA) is equivocal.^3 12–16^ In a recent systematic review, nine prospective cohort studies did not demonstrate an association between pincer morphology and RHOA, whereas cross-sectional studies showed that hips with OA were 3.7 times more likely to have a lateral centre edge angle (LCEA) ≥40°.^3^ However, substantial heterogeneity (I2 60%) in meta-analysis of the prospective studies was observed, making it difficult to draw conclusions from this review.^3^ Furthermore, study populations and how pincer morphology is defined and measured vary significantly across studies, compromising the reported associations.

Our aim is to perform an individual participant data (IPD) meta-analysis on the association between pincer morphology at baseline and the risk of developing RHOA within 4–8 years follow-up. Additionally, we will study this association in subgroups stratified by age, biological sex and body mass index (BMI).

## Methods

### Study design and participants

Participants were drawn from the Worldwide Collaboration on OsteoArthritis prediCtion for the Hip (World COACH) consortium. The World COACH consortium is a global collaboration of all available prospective cohort studies with prospective pelvic or hip imaging. The consortium profile has been published in detail elsewhere.^17^

In this study, we included all cohorts with a follow-up antero-posterior (AP) pelvic radiograph within 4–8 years of a baseline radiograph, and therefore included nine cohorts (Cohort Hip and Cohort Knee (CHECK), Multi-center Osteoarthritis Study (MOST), Osteo Arthritis Initiative (OAI), Rotterdam Study-I (RS-I), Rotterdam Study-II (RS-II), Rotterdam Study-III (RS-III), the Chingford Study, The Johnston County Project (JoCo) and the Study of Osteoporotic Fractures (SOF)) and excluded two cohorts (Tasmanian Older Adults Cohort (TASOAC), FAI and hip osteoarthritis cohort).

All included hips needed to have known BMI, biological sex and age at baseline. Hips without an original baseline RHOA score were excluded. Additionally, all radiographs of insufficient quality for automated pincer morphology measurements and all AP hip radiographs were excluded as they did not allow for constructing a horizontal reference line to adjust for pelvic rotation. Next, we excluded all hips lacking an original RHOA score at follow-up and excluded all baseline hips with acetabular dysplasia (AD) as determined by a Wiberg centre edge angle≤25°. We chose to do this in order to compare the pincer hips to a reference group of hips with normal acetabular coverage. Furthermore, multiple studies have demonstrated a significant association between AD and RHOA.^4 13 18^ Finally, we included only hips free of any signs of RHOA at baseline (any OA score=0). We chose to focus on a population of hips completely free of RHOA to identify the true predictors of this disease. This led to a total inclusion of 18 935 hips. Data missingness is described in online supplemental material 3.

### Radiographs

AP pelvic radiographs were obtained by cohorts at baseline and at follow-up between 4 and 8 years (online supplemental material 1). All radiographs were obtained based on a cohort-specific predetermined protocol established by each cohort. Detailed information about specific radiographic protocols was previously published.^17^ Five cohorts (CHECK, OAI, RS-I, RS-II and RS-III) had weight-bearing AP pelvic radiographs, one cohort (MOST) had weight-bearing full-limb radiographs, and three cohorts (the Chingford Study, JoCo and SOF) had supine AP pelvic radiographs.

### Radiographic measurements

#### Lateral centre edge angle

To avoid measurement variability across cohorts, uniform pincer morphology measurements were performed on all baseline radiographs. The bony outline of the proximal femur and acetabulum was annotated on the AP pelvic radiographs with a point set using the BoneFinder software (www.bone-finder.com; The University of Manchester, UK).^19^ This point set was used to perform automated radiographic measurements using a previously published Python script, which was adjusted and validated on World COACH data.^20 21^

**Figure 1:**
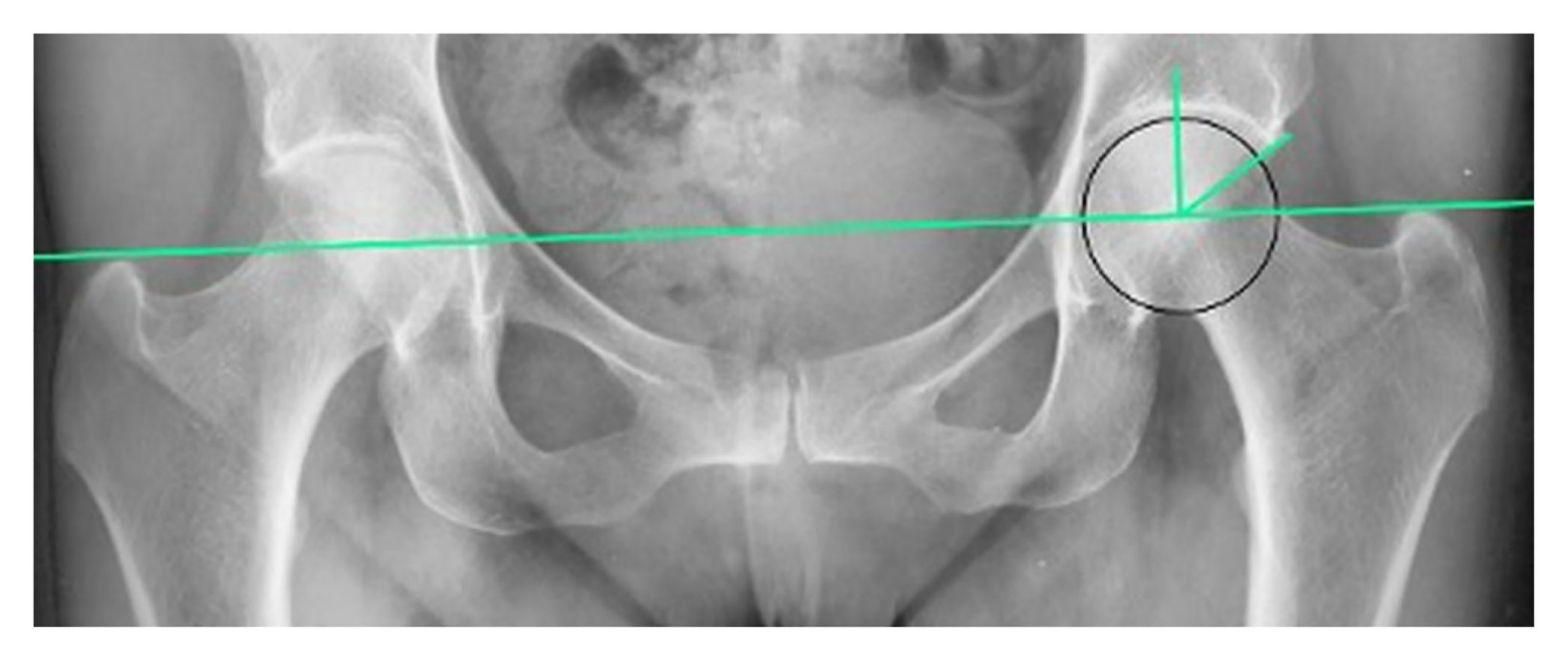
The lateral centre edge angle (LCEA) is measured on an anteroposterior (AP) pelvic radiograph. The LCEA was constructed according to the following steps. A horizontal reference line was constructed to correct for pelvic tilt in the radiograph and was based on the average of four lines, between (1) both femoral head centres, (2) the most cranial points of the foramen obturator, (3) the most caudal point of the ischial tuberosity and (4) the most caudal point of the pelvic teardrop. To determine the centre of the femoral head, a best fitting circle was drawn around the femoral head based on the statistical shape modeling (SSM) points. The LCEA was then formed by two lines drawn from the centre of the best fitting circle. The first line was drawn vertically through the centre of the femoral head, perpendicular to the horizontal reference line. The second line was drawn from the centre of the best fitting circle to the most lateral bony point of the acetabulum.

The LCEA quantifies bony coverage of the femoral head by the acetabulum (figure 1).^22^ Moderate pincer morphology was defined as an LCEA ≥40°. Sensitivity analyses with an LCEA threshold of ≥45° to define severe pincer morphology were performed to determine whether increased acetabular overcoverage influences the risk of developing RHOA.

#### RHOA grading

Original OA scores per cohort were harmonised into ‘free of RHOA’ (any score 0), ‘doubtful RHOA’ (any score 1) or ‘definite RHOA’ (KL ≥2, modified croft ≥2, modified OA=2 or total hip replacement).^23^

### Outcome measurements

The primary outcome was ‘definite RHOA’ defined by the harmonised RHOA score (OA score=2) within 4–8 years follow-up from baseline. Additionally, RHOA was defined as an ordinal outcome ‘free of RHOA’, ‘doubtful RHOA’ and ‘definite RHOA’ in secondary analyses.

### Statistical analysis

**Figure 2:**
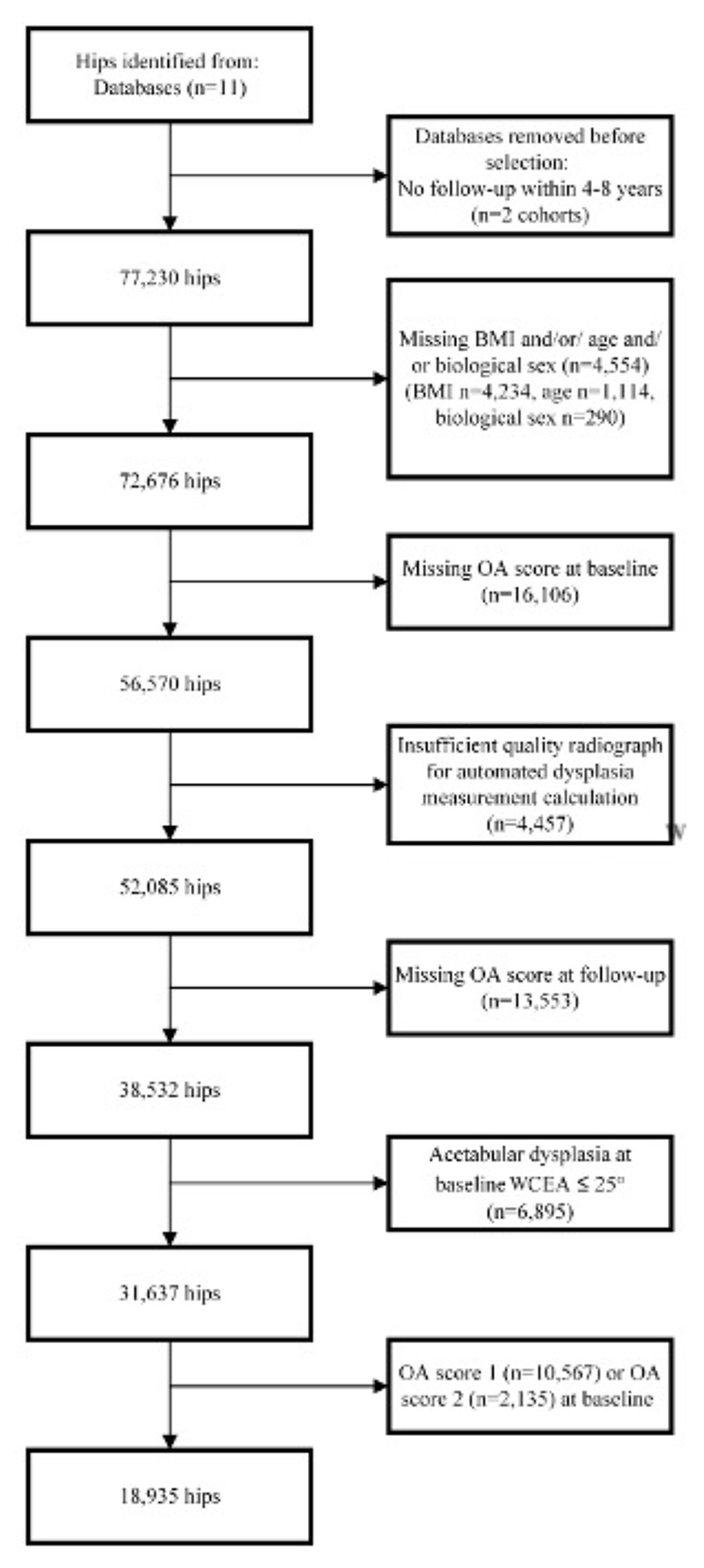
Flow of hips from consortium inclusion to final study population. BMI, body mass index; OA, osteoarthritis.

All statistical analyses were performed in R V.4.1.1. Univariate differences in baseline characteristics between complete included and excluded cases were inspected, meaning the included hips were compared with the hips that were excluded because of an OA score of 1 or 2 at baseline (figure 2). The association between baseline moderate pincer morphology defined by LCEA ≥40° and incident RHOA was estimated using a one-stage logistic regression model with generalised mixed effects with three levels: hip side (left/right), individual and cohort. We corrected for the cohort in this multilevel model in order to adjust for possible residual confounding by study differences. The model accounted for the difference between open (Chingford, JoCo, RS-I, RS-II, RS-III) and closed population cohorts (CHECK, OAI, MOST, SOF). The inclusion criteria for various population types vary notably, with a key distinction centred on enrolment characteristics. The results are expressed as adjusted OR (aOR) and unadjusted ORs with 95% CI and were adjusted for baseline age, modelled using splines with three df to account for non-linearity, biological sex and BMI. A sensitivity analysis was performed using LCEA ≥45° to define severe pincer morphology. In the sensitivity analysis, hips with a 40° ≤LCEA <45° were excluded from the reference group in order to compare pincer hips to a population of hips free of pincer morphology by any definition. The statistical significance threshold was set at p<0.05. To investigate the impact of the full-limb radiographs, which are traditionally not used to define hip morphology, on the association between pincer morphology and RHOA, we repeated the analyses excluding the MOST cohort. Additionally, a continuation ratio model with ordinal outcome RHOA classified as ‘free of RHOA’, ‘doubtful RHOA’ and ‘definite RHOA’ was created to assess the influence of doubtful RHOA as reference group. Random effects were added to adjust for clustering of cohorts and individuals, and the model was adjusted for baseline age, sex and BMI. Moderate pincer morphology was defined as LCEA ≥40°. The model was built in a forward fashion and a relaxed ordinality assumption for pincer morphology, allowing the effect of pincer morphology to be different for each level of the outcome RHOA within 4–8 years. The results were presented as an effect plot of the marginal probabilities with reference to the random effects for females, with mean baseline age and BMI and randomly selected left hip side. Due to limited outcomes, it was not possible to perform subgroup analyses using the same logistic regression model used in our primary analysis. Instead, we reported absolute risk (AR) and relative risk (RR) of developing RHOA in hips with moderate pincer morphology and non-pincer hips. These estimates were stratified by age (40–50, 51–60, 61–70 and >70 years of age), BMI (BMI >25 and BMI ≤25) and biological sex. The AR 95% CI was calculated based on the observed AR: AR±1.96*sqrt((AR(1−AR))/total number of individuals. The RR with corresponding 95% CI was determined by unconditional maximum likelihood estimation. No additional covariates were included in these stratified risk estimates. Logistic regression was performed using the lme4 package.^24^ The continuation ratio model was created using the GLMMadaptive package.^25^ The effect plot was created using the ggplot2 package.^26^

### Equity, diversity and inclusion statement

The current study includes participants with a variety of ethnic backgrounds, more women than men and individuals from three continents from high-income countries. Our study includes individuals from marginalised communities, including ethnic minorities, women, elderly and low-income and rural communities from high-income countries. Details are reported in the World COACH description paper.^17^ The author team is gender balanced and includes both junior and senior researchers with a variety of academic backgrounds who were actively involved in the writing process.

## Results

### Participants

The flow of World COACH hips to the current final study population is depicted (figure 2). 18 935 hips were included for analysis. The average time between the baseline and follow-up radiograph across all cohorts is 6.0±1.7 years. Baseline demographic data stratified per cohort are presented in table 1. The excluded hips were on average slightly older (65.68 years vs 62.66 years at baseline) and had a higher prevalence of pincer morphology as defined by moderate and severe thresholds.

### Pincer morphology

A total of 4894 (25.8%) hips had moderate pincer morphology defined by LCEA ≥40° and 1121 (5.9%) hips had severe pincer morphology defined by a threshold LCEA≥45°. In females, 3542 (26.6%) hips had moderate pincer morphology and 810 (6.1%) had severe pincer morphology. In males, 1352 (24.1%) hips had moderate pincer morphology and 311 (5.5%) severe pincer morphology.

### Incident RHOA

Definite RHOA had developed in 352 hips (1.9 %) within 8 years follow-up. The distribution of RHOA incidence per cohort is 82 hips (12.1%) in CHECK, 72 hips (8.8%) in Chingford, 54 hips (8.5%) in JoCo, 7 hips (0.6%) in MOST, 13 hips (0.3%) in OAI, 12 hips (0.5%) in RS-I, 6 hips (0.4%) in RS-II, 53 hips (2.2%) in RS-III and 53 (1.9%) in SOF.

### Association between pincer morphology and RHOA

The association between moderate pincer morphology and incident RHOA within 8 years was 1.15 (0.92–1.43), (p=0.22). The association between severe pincer morphology and incident RHOA was 1.50 (1.05–2.14), with a (p=0.026). The associations between pincer morphology and incident RHOA are summarised in table 2.

The marginal probability for hips with moderate pincer morphology (LCEA≥40°) to develop doubtful RHOA within 4–8 years is 0.20 (95% CI 0.14 to 0.28), compared with 0.17 (95% CI 0.11 to 0.24) for hips free of pincer morphology. The marginal probability for moderate pincer hips (LCEA≥40°) to develop definite RHOA within 4–8 years is 0.03 (95% CI 0.01 to 0.06), compared with 0.02 (95% CI 0.01 to 0.06) for pincer-free hips. The effect plot of the marginal probabilities from the continuation ratio model with ordinal outcome RHOA is shown in online supplemental material 2.

### Sensitivity analysis excluding the MOST cohort

The study population excluding the MOST cohort comprised a total of 17 733 hips. Of all hips in the study population, only seven hips developed RHOA within 8 years in the MOST cohort. No hips with pincer morphology developed RHOA. The non-significant association between hips with moderate pincer morphology and incident RHOA was 1.19 (95% CI 0.90 to 1.56, p=0.22) in the remaining study population (n=17 733) when hips from the MOST cohort were excluded.

### Subgroup analyses

Descriptive statistics stratified by age group, biological sex and BMI are summarised in table 3. The RR for moderate pincer hips to develop RHOA was highest in age group 40–50 (RR 2.66 (95% CI 1.43 to 4.95, p value 0.004), in hips with BMI ≥25 (RR 1.29 (95% CI 1.00 to 1.71), p value 0.078) and in female hips (RR 1.20 (95% CI 0.93 to 1.56), p value 0.16).

## Discussion

This first IPD meta-analysis in a large prospective consortium completely free of RHOA at baseline did not find a significant association between moderate pincer morphology defined by LCEA ≥40° and incident RHOA within 8 years. However, severe pincer morphology (LCEA≥45°) was significantly associated with RHOA. Hips with moderate pincer morphology may also be more likely to progress to doubtful RHOA within this follow-up compared with non-pincer hips, although no conclusions on clinical significance can be drawn. Subgroup statistics point in the direction that hips with moderate pincer morphology in younger individuals (aged 40–50) and with higher baseline BMI (≥25) are more at risk of developing RHOA compared with non-pincer hips. Additionally, hips in females with moderate pincer morphology were slightly more at risk to develop RHOA within 8 years compared with hips in males.

Several previous studies have been unable to establish an association between moderate pincer morphology and RHOA.^3^ A large prospective study of over 4000 hips with 9.2 years of follow-up from the Rotterdam Study found no significant association between pincer morphology, defined as an LCEA≥40°, and RHOA.^13^ Similarly, in the CHECK cohort, of 1002 hips with 10 years follow-up, no overall association was observed; however, the presence of hip pain at baseline did appear to modify this relationship, as acetabular overcoverage increased the risk of developing RHOA in these cases.^27^ In primary analyses, our study did not find a significant association between moderate pincer morphology and RHOA. Nevertheless, prior results from the Rotterdam Study demonstrated that pincer morphology increased the risk of developing RHOA specifically in hips that were completely free of RHOA at baseline (KL grade 0).^13^ Furthermore, a cross-sectional study by Faber *et al* reported that pincer morphology was associated with an increased risk of joint space narrowing, providing additional evidence that pincer morphology may pose a risk for developing RHOA.^16^ Interestingly, in secondary analyses of our study data, severe pincer morphology (LCEA ≥45°) was significantly associated with the development of RHOA, further emphasising the complexity of this relationship.

In the present study, the average BMI was 27.4 kg/m^2^. Conflicting evidence exists on the relationship between increased BMI and hip OA, although a systematic review suggested that the risk of hip OA increases with BMI in a dose–response relationship.^28^ In our study, in a subgroup of people with a baseline BMI ≥25 kg/m^2^, descriptive statistics indicated that pincer morphology hips—compared with non-pincer hips, had a higher RR (1.23 vs 0.79) of developing hip OA. Importantly, however, their CIs overlap.

Our study population consists mostly of female hips (70%), but the incidence of pincer morphology was similar in female and male hips (26.6% and 24.1%, respectively). This is in line with previous findings.^29^ Research shows that women have greater pelvic obliquity and less vertical centre of mass displacement compared with men, which may influence biomechanics of the hip joint, and could potentially lead to a higher RHOA risk in female hips with pincer morphology.^30^ Unfortunately, it was not possible to perform regression analyses in subgroups by biological sex in the present study, as only 19 male hips with pincer developed RHOA.

It is possible that the definition of pincer morphology has a direct impact on its association with RHOA.^29^ This is illustrated by the significant association between severe pincer morphology defined by LCEA≥45° and incident RHOA, which was not present in the current population when moderate pincer morphology was defined by LCEA≥40°. Most studies have relied on an LCEA ≥40° to define pincer morphology, but based on results from the present study with almost 19 000 hips, we argue that this threshold may be too low to be clinically relevant. A recent study of 6807 individuals from the UK Biobank found a prevalence in the general population of pincer morphology defined by an LCEA ≥45°, of 8.1% in females and 8.9% in males.^16^ This is similar to the prevalence in this study (LCEA≥45° 6.1% in female hips and 5.5% in male hips). In the excluded hips from the present study, severe pincer morphology prevalence was 14.3%. These hips were only excluded from analysis because they were not free of RHOA at baseline. It may be that these hips had already developed RHOA as a result of acetabular overcoverage. This is further supported by the increased RR in the younger subgroups as pincer morphology may be a considerable risk factor for more rapid development of RHOA. On the other hand, the LCEA might also be influenced by the presence of RHOA, for example, due to (subtle) acetabular osteophytes, which potentially cause false-positive classification of pincer morphology. This is why we excluded these hips. Subsequent studies should aim to conduct sensitivity analyses employing this threshold, which may elucidate a more clinically relevant study population in the search for modifiable risk factors for RHOA.

Importantly, the definition of pincer morphology as a static concept (defined only by radiological excessive acetabular coverage) differs from the dynamic concept of FAI syndrome with pincer morphology. The definition of FAI syndrome, as stated by the 2016 Warwick Agreement, does not only pertain to radiological findings, but to a triad of radiological signs, clinical signs (hip impingement tests, limited range of motion) and symptoms (motion or position-related pain in the hip or groin).^10^ Clinical signs and symptoms have not yet been harmonised in the world COACH consortium but including those might enhance the predictive ability and identify hips with pincer morphology, which are at higher risk of developing RHOA as this has been shown previously for patients with FAI syndrome with cam morphology in the CHECK cohort.

This study has several strengths. First, we included hips completely free of any signs of RHOA at baseline, which differs from some previous prospective studies.^3 4 13^ This allowed us to study associations that were unbiased by pre-existing doubtful RHOA. Though previous prospective studies generally corrected for baseline RHOA grade in statistical models, we believe risk factors are best demonstrated when RHOA-free hips are followed until a subset develops disease. Furthermore, LCEA measurements may be affected by the presence of osteophytes as it is possible that spurious osteophytes are mistaken for pincer hips. We were able to rule out the presence of osteophytes at baseline as all included hips were completely free of RHOA. Second, IPD meta-analysis study design is a significant strength. By collecting, pooling and analysing original cohort data, we achieved increased statistical power allowing for subgroup and sensitivity analyses. Our results confirm a robust estimate of the risk pincer morphology poses to RHOA-free hips within 8 years. This could inform the clinical approach to patients with severe pincer morphology, including future treatment and preventative strategies for hip OA. Finally, we used uniform automated measurements. Using a validated algorithm to quantify acetabular coverage of all hips on baseline radiographs reduces variability and bias in predictor measurements.

### Limitations

This study is subject to several limitations. First, it has been suggested that pincer morphology potentially only leads to RHOA when mixed with other shape features, or specific subtypes of pincer morphology which were not captured by the LCEA only.^31^ The LCEA, however, is presently the most commonly used and reliable measurement of pincer morphology.^32^ Furthermore, a recent study compared radiographs to CT scans and found similar sensitivity and specificity in defining pincer morphology when comparing radiographs to CT scans.^33^ Second, we included both supine and weight-bearing radiographs, which may influence RHOA grading. However, a study comparing the joint space width (JSW) on weight-bearing and supine radiographs found that how the radiograph was obtained does not significantly impact JSW measurements.^34^ In the present study, we adjusted for age, biological sex and BMI as these are important in hip OA research and were harmonised variables in the world COACH consortium.^17^ Importantly, unmeasured confounders that were not incorporated may be important in the association of pincer morphology and hip OA and should be studied in future work. Additionally, although the World COACH consortium includes a large and diverse sample, all cohorts are based in high-income countries. Given that low-income and middle-income countries also bear a substantial burden of hip OA, the generalisability of our findings to these populations may be limited. Finally, we only studied RHOA, which may differ from clinically relevant hip OA where symptoms are taken into account. Elucidating the association between pincer morphology and a clinical definition of hip OA should be prioritised in future research.

Modifiable risk factors are essential for preventing hip OA in the general population as well as athletes. Our study shows that severe pincer morphology only explains a small subset of individuals at risk for hip OA. However, we argue that severe pincer morphology is a potentially modifiable risk factor for hip OA for at least three reasons. First, physical therapy might increase strength and stability of the joint. Second, activity modification might help avoid excessive joint loading. Third, surgical interventions might help improve the joint shape and could potentially aid in preventing OA, although this is presently unknown. Prevention of hip OA can improve overall quality of life and aid in relieving the substantial and increasing societal burden of this disease.^35^

To the best of our knowledge, our IPD meta-analysis is the first study of its kind to investigate the relationship between pincer morphology and the risk of developing RHOA. Severe pincer morphology defined by an LCEA ≥45° is significantly associated with incident RHOA in a population of RHOA-free hips at baseline. This study offers new insights into a potentially modifiable risk factor for RHOA in specific subgroups, which contributes to discovering targets for prevention and treatment of hip OA in the future.

### Clinical implications

Pincer morphology, characterised by acetabular over-coverage, is not currently identified as a risk factor for RHOA in the existing literature, potentially due to variability in measurement methods, thresholds and study populations as well as reader variability in manual measurements. However, this study provides robust evidence that severe pincer morphology, defined by a LCEA of 45° or more, is significantly associated with incident RHOA, whereas a LCEA of 40° is not. By including only hips free of RHOA at baseline and accounting for variations in defining the outcome, this analysis avoids inconsistencies seen in prior research. Since OA significantly impacts quality of life, understanding how pincer morphology increases the risk of disease could help identify high-risk individuals and inform strategies to mitigate this modifiable risk factor.

## Figures and Tables

**Table 1:** Baseline characteristics of included hips, stratified per cohort

	CHECK	Ching	JoCo	MOST	OAI	RS-I	RS-II	RS-III	SOF	Total incl.	Total excluding*
Hips, n (%)	678	815	633	1202	4481	2719	1797	2381	4229	18 935	12 702
Age, mean (SD) years	55.64 (5.28)	53.62 (5.73)	59.25 (8.72)	61.09 (7.24)	60.66 (8.92)	65.21 (6.33)	63.00 (6.40)	56.34 (4.85)	70.38 (4.36)	62.66 (8.36)	65.68 (8.32)
BMI, mean (SD) kg/m^2^	26.29 (4.23)	25.38 (3.97)	29.60 (6.02)	29.93 (5.13)	28.27 (4.64)	26.15 (3.45)	27.1 1 (3.83)	27.56 (4.17)	26.38 (4.31)	27.30 (4.49)	27.51 (4.75)
Men, n (%)	114 (16.8)	0 (0.0)	282 (44.5)	378 (31.4)	1860 (41.5)	1120 (41.2)	795 (44.2)	1065 (44.7)	0 (0)	5614 (29.6)	3444 (27.1)
Moderate pincer morphology (LCEA≥40°), n (%)	143 (21.1)	261 (32.0)	149 (23.5)	221 (18.4)	1014 (22.6)	887 (32.6)	440 (24.5)	522 (21.9)	1257 (29.7)	4894 (25.8)	5099 (40.1)
Severe pincer morphology (LCEA≥45°), n (%)	33 (4.9)	73 (9.0)	35 (5.5)	55 (4.6)	204 (4.6)	230 (8.5)	93 (5.2)	108 (4.5)	290 (6.9)	1121 (5.9)	1815 (14.3)
OAscore=2 follow-up, male/female (%/%)	19/63 (16.7/11.2)	0/72 (0.0/8.8)	18/36 (6.4/10.3)	3/4 (0.8/0.5)	3/10 (0.2/0.4)	1/11 (0.1/0.7)	2/4 (0.3/0.4)	36/17 (3.4/1.3)	0/53 (0.0/1.3)	82/270 (1.5/2.0)	3025

OA score: 0= no RHOA, 1= doubtful RHOA, 2= definite RHOA.

* Excluded hips are defined as all eligible hips for analysis but with OA score 1 or 2 at baseline. CHECK, Cohort Hip and Cohort Knee; Ching, the Chingford Study; JoCo, The Johnston County Project; LCEA, lateral centre edge angle; LCEA, Lateral Center Edge Angle; MOST, Multi-center Osteoarthritis Study; OA, osteoarthritis; OAI, Osteo Arthritis Initiative; RHOA, radiographic hip osteoarthritis; RS-I, Rotterdam Study-I; RS-II, Rotterdam Study-II; RS-III, Rotterdam Study-III (RS-III); SOF, Study of Osteoporotic Fractures

**Table 2:** Associations between severe and moderate pincer morphology and RHOA

Definition pincer morphology	Hips with pincer morphology (%)	Hips with incident RHOA at follow-up (%)	Absolute risk (%)	Unadjusted OR (95% CI)	P value	Adjusted OR (95% CI)*	P value
Moderate pincer morphology (LCEA≥40°)	4894	101	101/4894 (2.1)	1.19 (0.91 to 1.57)	0.21	1.15 (0.92 to 1.43)	0.22
Severe pincer morphology (LCEA≥45°)	1121	31	31/1121 (2.8)	1.57 (1.10 to 2.24)	0.013	1.50 (1.05 to 2.14)	0.026

* Adjusted for age, biological sex and body mass index (BMI) LCEA, lateral centre edge angle; RHOA, radiographic hip osteoarthritis.

**Table 3:** Absolute and relative risk of hips with moderate pincer morphology to develop incident radiographic hip osteoarthritis stratified by age group, BMI and biological sex

Strata	Total hips in group, n	Hips with moderate pincer morphology (LCEA≥40°), n (%)	Hips with incident RHOA, n (%)	Hips with moderate pincer morphology (LCEA≥40°), and incident RHOA, n	Absolute risk, % (95% CI) *	Relative risk, % (95% CI) †	P value
Age (years)							
40–50	1534	307 (20.0)	40 (2.6)	16	1.0 (0.5 to 1.6)	2.66 (1.43 to 4.95)	0.004
51–60	6180	1363 (22.1)	149 (2.4)	47	0.8 (0.5 to 1.0)	1.63 (1.16 to 2.29)	0.007
61–70	7557	2056 (27.2)	110 (1.5)	26	0.3 (0.2 to 0.5)	0.83 (0.53 to 1.28)	0.45
70+	3664	1168 (31.9)	53 (1.4)	12	0.3 (0.1 to 0.5)	0.62 (0.32 to 1.19)	0.18
BMI (*kg/m^2^***)**							
<25	6094	1600 (26.3)	125 (2.1)	31	0.5 (0.3 to 0.7)	0.93 (0.62 to 1.38)	0.76
≥25	12 841	3294 (25.7)	227 (1.8)	70	0.5 (0.4 to 0.7)	1.29 (1.00 to 1.71)	0.078
Biological sex							
Male	5614	1352 (24.1)	82 (1.5)	19	0.3 (0.2 to 0.5)	0.95 (0.57 to 1.58)	1
Female	13 321	3542 (26.6)	270 (2.0)	82	0.6 (0.5 to 0.7)	1.20 (0.93 to 1.56)	0.16

* The absolute risk was calculated using the following equation: (number of hips with pincer morphology and RHOA/total number of hips in subgroup).

† The relative risk was calculated using the following equation: (number of hips with pincer and RHOA/(number of hips with pincer and RHOA+number of hips with pincer only))/(number of hips with RHOA without pincer morphology/(number of hips with RHOA without pincer morphology+number of hips without pincer morphology or RHOA)).

BMI, body mass index; LCEA, lateral centre edge angle; RHOA, radiographic hip osteoarthritis.

## Data Availability

Data are available upon reasonable request. Data may be obtained from a third party and are not publicly available. We encourage the use of data by third parties, although this is subject to approval by the steering committees of the World COACH consortium and the participating cohorts, as well as to legal boundaries regarding data ownership. A standardised data request form is available for which will be reviewed uniformly in order to consistently handle World COACH data requests.

## What is Already Known on this Topic

**⇒**
Pincer morphology, characterised by acetabular overcoverage of the femoral head, which leads to impingement, has been proposed as a risk factor for hip osteoarthritis (OA). Evidence for the association between pincer morphology and hip OA, however, remains conflicting.

## What are the Findings?

**⇒**
This study provides evidence that severe pincer morphology (lateral centre edge angle ≥45°) in hips free of radiographic hip OA at baseline is significantly associated with incident radiographic hip OA at 4–8 years follow-up.

## How Might it Impact on Clinical Practice in the Future?

**⇒**
⇒ Given the substantial impact of OA on quality of life, elucidating the role of pincer morphology in OA risk may help clinicians to identify at-risk individuals and guide preventive interventions.
